# Different phenotypes of neuropsychiatric systemic lupus erythematosus are related to a distinct pattern of structural changes on brain MRI

**DOI:** 10.1007/s00330-021-07970-2

**Published:** 2021-04-30

**Authors:** Francesca Inglese, Ilse M. J. Kant, Rory C. Monahan, Gerda M. Steup-Beekman, Tom W. J. Huizinga, Mark A. van Buchem, Cesar Magro-Checa, Itamar Ronen, Jeroen de Bresser

**Affiliations:** 1 Department of Radiology, Leiden University Medical Center (LUMC), Albinusdreef 2, 2333 ZA Leiden, The Netherlands; 2 Department of Radiology, University Medical Center Utrecht, Heidelberglaan 100, 3584 CX Utrecht, The Netherlands; 3 Department of Rheumatology, Leiden University Medical Center (LUMC), Albinusdreef 2, 2333 ZA Leiden, The Netherlands; 4 Department of Rheumatology, Zuyderland Medical Center, Henri Dunantstraat 5, 6419 PC Heerlen, The Netherlands

**Keywords:** Lupus erythematosus, Systemic, Phenotype, Magnetic resonance imaging, Brain

## Abstract

**Objectives:**

The underlying structural brain correlates of neuropsychiatric involvement in systemic lupus erythematosus (NPSLE) remain unclear, thus hindering correct diagnosis.

We compared brain tissue volumes between a clinically well-defined cohort of patients with NPSLE and SLE patients with neuropsychiatric syndromes not attributed to SLE (non-NPSLE). Within the NPSLE patients, we also examined differences between patients with two distinct disease phenotypes: ischemic and inflammatory.

**Methods:**

In this prospective (May 2007 to April 2015) cohort study, we included 38 NPSLE patients (26 inflammatory and 12 ischemic) and 117 non-NPSLE patients. All patients underwent a 3-T brain MRI scan that was used to automatically determine white matter, grey matter, white matter hyperintensities (WMH) and total brain volumes. Group differences in brain tissue volumes were studied with linear regression analyses corrected for age, gender, and total intracranial volume and expressed as *B* values and 95% confidence intervals.

**Results:**

NPSLE patients showed higher WMH volume compared to non-NPSLE patients (*p* = 0.004). NPSLE inflammatory patients showed lower total brain (*p* = 0.014) and white matter volumes (*p* = 0.020), and higher WMH volume (*p* = 0.002) compared to non-NPSLE patients. Additionally, NPSLE inflammatory patients showed lower white matter (*p* = 0.020) and total brain volumes (*p* = 0.038) compared to NPSLE ischemic patients.

**Conclusion:**

We showed that different phenotypes of NPSLE were related to distinct patterns of underlying structural brain MRI changes. Especially the inflammatory phenotype of NPSLE was associated with the most pronounced brain volume changes, which might facilitate the diagnostic process in SLE patients with neuropsychiatric symptoms.

**Key Points:**

*• Neuropsychiatric systemic lupus erythematosus (NPSLE) patients showed a higher WMH volume compared to SLE patients with neuropsychiatric syndromes not attributed to SLE (non-NPSLE).*

*• NPSLE patients with inflammatory phenotype showed a lower total brain and white matter volume, and a higher volume of white matter hyperintensities, compared to non-NPSLE patients.*

*• NPSLE patients with inflammatory phenotype showed lower white matter and total brain volumes compared to NPSLE patients with ischemic phenotype.*

**Supplementary Information:**

The online version contains supplementary material available at 10.1007/s00330-021-07970-2.

## Introduction

Systemic lupus erythematosus (SLE) is an autoimmune disease characterized by the production and deposition of autoantibodies and involvement of different organs, such as the kidneys, lungs, joints, skin, and also the brain. Neuropsychiatric (NP) symptoms are common in patients with SLE and can be directly associated with the disease (NPSLE) or can be explained by another etiology, such as side effects of medication or involvement of other organs (non-NPSLE) [1]. NPSLE is associated with an increased mortality and reduced quality of life within the SLE population [2]. The attribution of NP manifestations in SLE patients remains a challenge for clinicians as there are no (radiological or other) biomarkers for establishing a diagnosis and guiding therapy decision [3].

In clinical practice, therapeutic approach in NPSLE is based on the severity of symptoms and the suspected underlying pathophysiologic mechanism: inflammatory or ischemic [3]. The inflammatory pathway is thought to be caused by production of inflammatory mediators as well as increased permeability of the blood-brain barrier, leading to focal (e.g., seizure) and diffuse NP manifestations (e.g., psychosis) [4]. The ischemic pathway is thought to be caused by injury of large- or small-caliber vessels or by immune system activation, often leading to focal (e.g., stroke) and diffuse NP events (e.g., cognitive dysfunction) [4].

Several studies have shown that patients with SLE have more white matter hyperintensities (WMH) and to a lesser extent more atrophy and infarcts compared to controls [5–9]. In one study, out of 74 active NPSLE patients, 49% showed focal WMH [5]. These studies suggest that brain MRI markers might help in establishing NPSLE diagnosis. Brain MRI volumes have not yet been investigated in different clinical phenotypes of NP symptoms in SLE. Our hypothesis is that the pattern of structural brain changes, represented by brain volumes, is different across SLE phenotypes and that these different patterns may be used as potential biomarkers to improve the diagnostic procedure in these patients. We, therefore, aimed to assess brain tissue volume differences between patients with NPSLE and non-NPSLE and between different NPSLE phenotypes in a well-defined patient cohort.

## Materials and methods

### Study population

The LUMC is the national referral center for SLE patients with NP symptoms in The Netherlands. All patients come to the clinic for a 1-day visit and undergo a standardized evaluation that includes a combination of multidisciplinary medical assessments and extensive complementary tests including a brain MRI scan [10, 11]. This evaluation is followed by a multidisciplinary consensus meeting to decide whether the NP events are attributable to SLE based on (amongst others) the following factors: time between diagnosis of SLE and occurrence of neuropsychiatric symptoms, the type of symptoms and favoring factors or alternative diagnoses [12, 13]. If NPSLE is considered present, a consensus is reached regarding the suspected underlying pathophysiology of NPSLE: inflammatory or ischemic [14], based on radiological, serological, and clinical data. Depending on the phenotype, either immunosuppressant therapy or anticoagulant therapy is initiated [15]. This multidisciplinary process has been described in detail previously [10, 11]. The Leiden-The Hague-Delft ethics approval committee approved the study and all included patients signed informed consent.

In our study, 216 consecutive patients, with the clinical diagnosis of SLE referred to the Leiden NPSLE clinic between May 2007 and April 2015, were included. Of these, 28 patients were excluded because of uncertainty regarding NPSLE diagnosis, 8 for misdiagnosis established during follow-up visit, 3 patients were excluded because of motion artifacts in the MRI scans, 20 patients were excluded because of brain infarcts over 1.5 cm that hinder accurate brain volume measurements, and 2 patients were excluded due to the presence of other diseases (brain tumor and large arachnoid cyst). This resulted in a total of 155 patients (age range: 16 to 79 years) included in the present study. This patient group consisted of 38 NPSLE patients (mean age 40 years) and 117 non-NPSLE patients (mean age 42 years). Within the NPSLE patients, 26 had an inflammatory phenotype (mean age 39 years) and 12 had an ischemic phenotype (mean age 41 years).

### Clinical variables

Information on gender, age, cardiovascular risk factors (hypertension, smoking, body mass index (BMI), and diabetes), and SLE disease duration were obtained via interview with the patient and by studying medical records.

SLE activity and damage indices were scored for each patient: the SLE disease activity was determined using the Systemic Lupus Erythematosus Disease Activity Index 2000 (SLEDAI-2K) [16]; SLE irreversible damage was assessed with the Systemic Lupus International Collaborating Clinics/American College of Rheumatology Damage Index (SDI) [17].

### MRI protocol

All participants were scanned on a Philips Achieva 3-T MRI scanner (Philips Healthcare) equipped with a body transmit RF coil and an 8-channel receive head coil array. Patients were scanned according to a standardized scanning protocol. The sequences used for this project were as follows: a 3D T_1_-weighted scan (voxel size = 1.17 × 1.17 × 1.2 mm^3^; TR/TE = 9.8/4.6 ms) and a fluid-attenuated inversion recovery (FLAIR) scan. In total, 102 data sets included a 2D multislice FLAIR sequence (voxel size = 1.0 × 1.0 × 3.6 mm^3^; TR/TE/TI = 10,000/120/2800 ms) and 53 data sets included a 3D FLAIR (voxel size = 1.10 × 1.11 × 0.56 mm^3^; TR/TE/TI = 4800/576/1650 ms). The change in FLAIR protocol occurred in February 2013 (the year they switched from 2D to 3D).

### Image processing

**Fig. 1:**
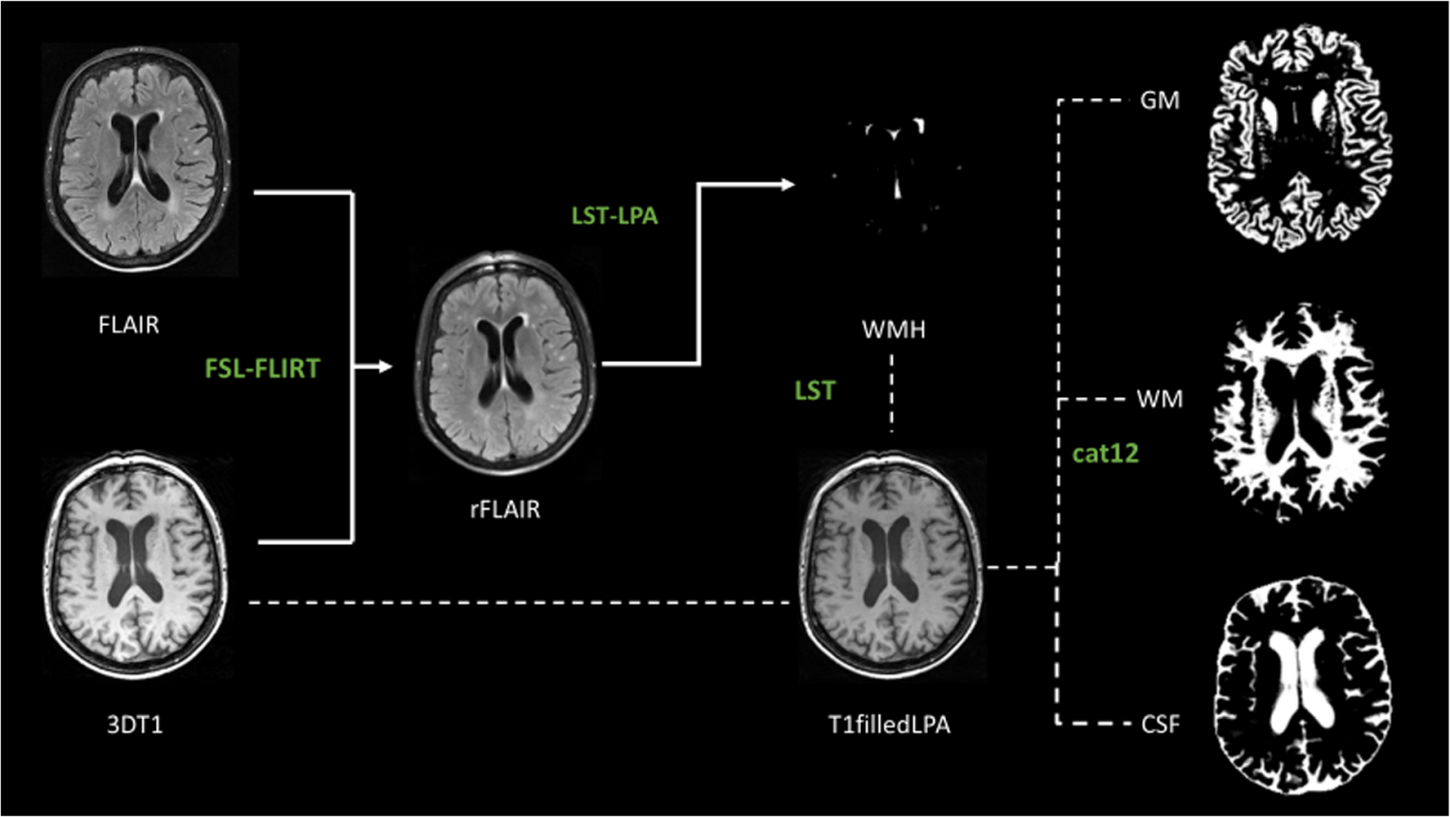
Image processing pipeline. The white arrows show the image processing pipeline to generate the white matter hyperintensities (WMH) segmentation. The dotted lines show the image processing pipeline to determine grey matter (GM), white matter (WM), and cerebral-spinal fluid (CSF) volumes. In green, the names of the used software: Linear Image Registration Tool (FLIRT) from the FMRIB Software Library v5.0 (FSL); lesion prediction algorithm (LPA), a toolbox of the Lesion Segmentation Toolbox (LST) version 2.0.15 for the statistical parametric mapping software (SPM12); the CAT12 toolbox from SPM12

FLAIR images were registered to the 3D T_1_-weighted images by using the Linear Image Registration Tool (FLIRT) from the FMRIB Software Library v5.0 [18, 19]. WMH segmentations were performed on the FLAIR images after registration to the T_1_-weighted images to generate WMH probability maps. These were performed using the lesion prediction algorithm [20], a toolbox of the Lesion Segmentation Toolbox version 2.0.15 for the statistical parametric mapping software. A lesion probability threshold of 0.2 was applied to the WMH probability maps to generate WMH masks, subsequently used to perform lesion filling on the 3D T_1_-weighted images with the lesion segmentation toolbox. This threshold was chosen after testing different thresholds between 0.1 and 0.5 on a random selection of patients where a threshold of 0.2 resulted in the best visual performance of WMH segmentation accuracy. The resulting lesion-filled 3D T_1_-weighted images were segmented using the CAT12 toolbox from the statistical parametric mapping software to determine total grey matter, white matter, and cerebral-spinal fluid volumes [21]. Total intracranial volume was calculated as the sum of grey matter, white matter, and cerebral-spinal fluid volumes. Total brain volume was determined as the sum of grey matter and white matter volumes. Figure 1 shows the image processing pipeline.

All MRI images as well as the grey matter, white matter, WMH, and cerebral-spinal fluid maps were visually inspected for segmentation errors and artifacts by a trained researcher (F.I.) and a neuroradiologist with 14 years of experience in brain segmentation (J.B.), both blinded to the clinical data.

### Statistical analysis

All statistical analyses were performed using IBM Statistical Package for the Social Sciences version 25. All data were tested for normal distribution by histograms visualization, q-q plot, and by calculating skewness and kurtosis values.

Differences in baseline characteristics between the NPSLE patients and non-NPSLE patients for nominal variables were determined with the chi-square test, and for continuous variables they were determined with an unpaired *t*-test or Mann-Whitney *U* test. Differences in grey matter, white matter, total brain, and WMH volumes between patients with NPSLE and non-NPSLE patients were assessed with linear regression analyses corrected for age, gender, and total intracranial volume. In secondary analyses, these linear regression analyses were additionally adjusted for hypertension, diabetes, smoking, BMI, and SLE duration (since the patients had SLE for many years at the time of inclusion).

Differences in baseline characteristics between the NPSLE patients with inflammatory phenotype and NPSLE patients with ischemic phenotype for nominal variables were determined with the chi-square test and for continuous variables they were determined with an unpaired *t*-test or Mann-Whitney *U* test. Differences in grey matter, white matter, total brain, and WMH volumes between NPSLE patients with inflammatory or ischemic phenotype and non-NPSLE patients and between NPSLE patients with inflammatory phenotype and NPSLE patients with ischemic phenotype were assessed with linear regression analyses corrected for age, gender, and total intracranial volume. In secondary analyses, these linear regression analyses were additionally adjusted for presence of hypertension, diabetes, smoking, BMI, and SLE duration.

Because of the non-normal distribution of the WMH volumes, they were multiplied by 1,000,000 and the natural logarithm of the resulting numbers was taken for the regression analyses.

## Results

**Fig. 2:**
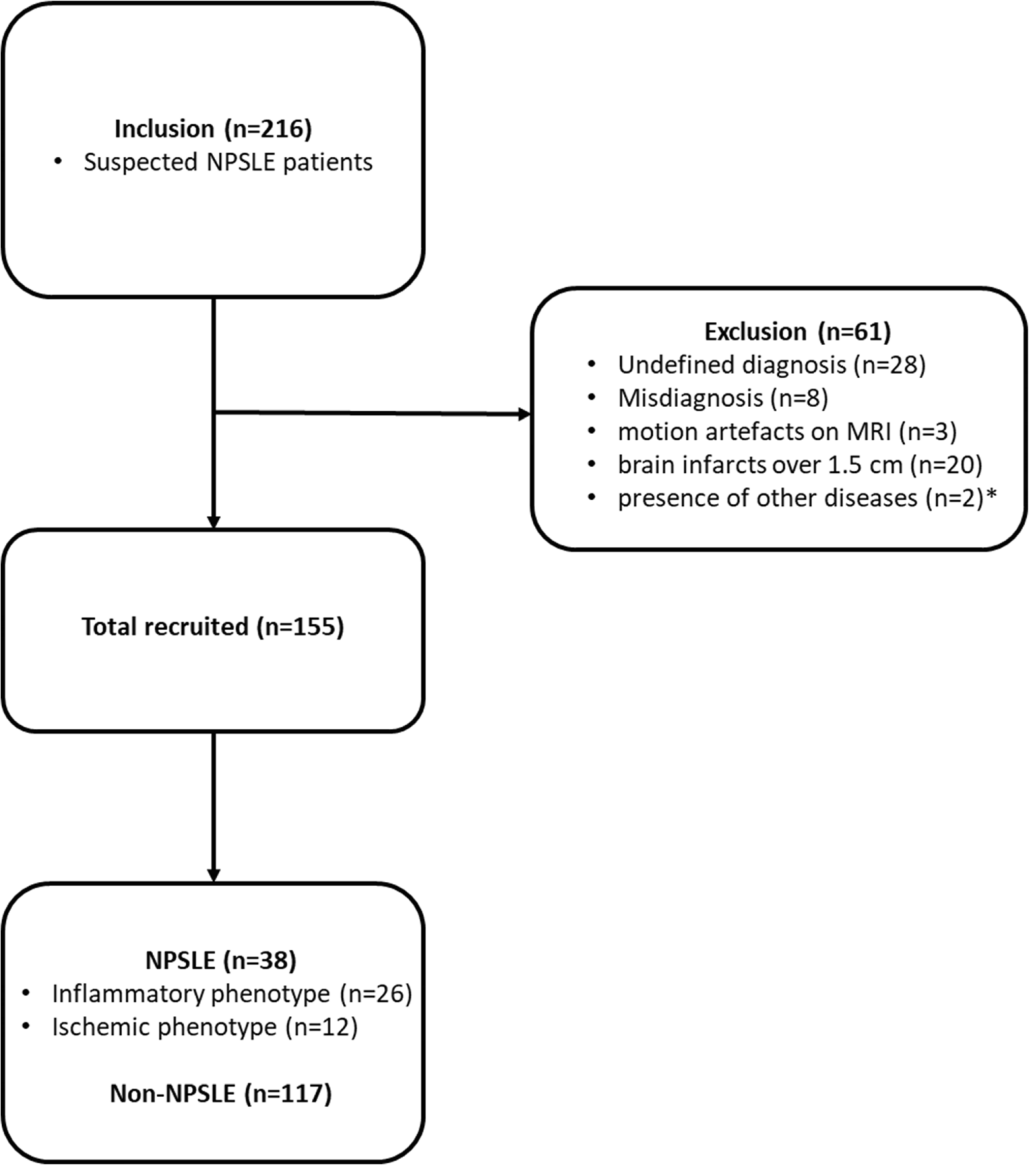
Flow diagram participants. One patient was excluded for the presence of a brain tumor and one for the presence of a large arachnoid cyst (indicated with an asterisk symbol). NPSLE, neuropsychiatric systemic lupus erythematosus; Non-NPSLE, non-neuropsychiatric systemic lupus erythematosus

A total of 155 SLE patients were included in the study of whom 38 NPSLE patients and 117 non-NPSLE patients. The inclusion and exclusion procedure is shown in Fig. 2.

Table 1 shows the characteristics of the NPSLE patients (*n* = 38; mean age 40 years; 87% female) and non-NPSLE patients (*n* = 117; mean age 42 years; 92% female). Compared to non-NPSLE patients, NPSLE patients showed a significantly higher SLEDAI-2K (*p* = 0.002) and SDI (*p* = 0.045), representing a higher disease activity and more irreversible damage. There were no between-group differences in cardiovascular risk factors (*p* > 0.05). Additional clinical variables are shown in Supplementary Tables 1 and 2.

**Table 1:** Patient characteristics of the NPSLE and non-NPSLE patients

	NPSLE patients (*n* = 38)	Non-NPSLE patients (*n* = 117)	*p* value
Female, *n* (%)	33 (87%)	108 (92%)	0.307
Age, years	40 ± 14	42 ± 13	0.351
Hypertension	16 (42%)	39 (33%)	0.326
Current smoking	5 (13%)	16 (14%)	0.720
BMI	25 ± 5	25 ± 4	0.990
Diabetes	3 (7%)	6 (5%)	0.626
Duration of SLE, years	6 ± 8	8 ± 8	0.083
SLEDAI-2K	8 ± 8	4 ± 4	0.002*
SDI	1.1 ± 1.1	0.8 ± 1.1	0.045*

Data represents *n* (percentage) or means ± standard deviations. Gender, age, cardiovascular risk factors, duration of disease, and damage indexes are shown. Differences between the two groups are expressed in *p* value and calculate for nominal variables with chi-square (gender, hypertension, smoking, and diabetes) and for continuous variables with an unpaired *t*-test (age and BMI) or Mann-Whitney *U* test (duration of SLE, SLEDAI-2K, and SDI) based on their distribution

*BMI* body mass index, *SLEDAI-2K* Systemic Lupus Erythematosus Disease Activity Index 2000, *SDI* Systemic Lupus International Collaborating Clinics Damage Index

*p < 0.05

Patients with NPSLE showed a significantly higher WMH volume compared to non-NPSLE patients ((*B* (95% CI): 0.67 (0.21 to 1.13)); *p* = 0.004). There were no statistically significant differences in white matter ((*B* (95% CI): −11.5 (−23.5 to 0.6)); *p* = 0.063), grey matter ((*B* (95% CI): −8.3 (−20.3 to 3.6)); *p* = 0.170), and total brain volume ((*B* (95% CI): −19.8 (−40.5 to 0.9)); *p* = 0.061) between the patients with NPSLE and patients with non-NPSLE (Table 2). These results did not attenuate in secondary analyses that were additionally adjusted for hypertension, diabetes, smoking, BMI, and SLE duration (Supplementary Table 3).

**Table 2:** Differences in brain volumes between the NPSLE patients and the non-NPSLE patients

	NPSLE patients (*n* = 38)	Non-NPSLE patients (*n* = 117)	NPSLE vs non-NPSLE (*B* (95% CI))
White matter volume	473 ± 55	482 ± 57	−11.5 (−23.5 to 0.6)
Grey matter volume	557 ± 62	560 ± 61	−8.3 (−20.3 to 3.6)
Total brain volume	1030 ± 110	1042 ± 111	−19.8 (−40.5 to 0.9)
WMH volume	1.08 (0.12–14.94)	0.60 (0.11–5.04)	0.67 (0.21 to 1.13)*

The second and third columns represent white matter, grey matter, total brain, and white matter hyperintensity (WMH) volume in ml expressed as means ± standard deviations or median (10–90% confidence intervals)

The fourth column represents *B* (95% confidence intervals) of the linear regression analyses on brain and WMH volumes in NPSLE patients versus non-NPSLE patients, adjusted for gender, age, and total intracranial volume. For the linear regression analysis, the WMH were multiplied by 1,000,000 and natural log transformed, because of non-normal distribution

*p < 0.05

Table 3 shows the characteristics of the NPSLE cohort subdivided into the inflammatory (*n* = 26; mean age 39 years; 89% female) and ischemic (*n* = 12; mean age 41 years; 83% female) phenotypes. NPSLE patients with inflammatory phenotype had a significantly higher disease duration (*p* = 0.012) and a higher SLEDAI-2K score (*p* = 0.030) compared to NPSLE patients with ischemic phenotype. There were no differences between groups in cardiovascular risk factors (*p* > 0.05).

**Table 3:** Patient characteristics of the NPSLE patients with an inflammatory phenotype and the NPSLE patients with an ischemic phenotype

	NPSLE inflammatory (*n* = 26)	NPSLE ischemic (*n* = 12)	*p* value
Female, *n* (%)	23 (89%)	10 (83%)	0.664
Age, years	39 ± 15	41 ± 11	0.581
Hypertension	11 (42%)	5 (42%)	0.970
Current smoking	3 (12%)	2 (17%)	0.666
BMI	24 ± 5	26 ± 5	0.274
Diabetes	1 (3%)	2 (15%)	0.154
Duration of SLE	4 ± 6	10 ± 10	0.012*
SLEDAI-2K	10 ± 9	4 ± 3	0.030*
SDI	1.0 ± 1.1	1.3 ± 1.0	0.185

Data represents *n* (percentage) or means ± standard deviations. Gender, age, cardiovascular risk factors, duration of disease, and damage indexes are shown. Differences between the two groups are expressed in *p* value and calculate for nominal variables with chi-square (gender, hypertension, smoking, and diabetes) and for continuous variables with an unpaired *t*-test (age and BMI) or Mann-Whitney *U* test (duration of SLE, SLEDAI-2K, and SDI) based on their distribution

*BMI* body mass index, *SLEDAI-2K* Systemic Lupus Erythematosus Disease Activity Index 2000, *SDI* Systemic Lupus International Collaborating Clinics Damage Index

*p < 0.05

NPSLE patients with inflammatory phenotype showed a significantly lower white matter (*B* (95% CI): −17.1 (−31.4 to −2.8); *p* = 0.020) and total brain volume (*B* (95% CI): −30.7 (−55 to −6.4); *p* = 0.014) and a higher WMH volume (*B* (95% CI): 0.87 (0.33 to 1.41); *p* = 0.002), but no statistically significant difference in grey matter volume (*B* (95% CI): −13.7 (−27.6 to 0.3); *p* = 0.055) compared to patients with non-NPSLE. These results did not attenuate when analyses were additionally adjusted for hypertension, diabetes, smoking, BMI, and SLE duration (Supplementary Table 4), but the difference in grey matter volume went to a statistically significant difference (*B* (95% CI): −14.8 (−29.1 to −0.5); *p* = 0.042). There were no significant differences observed in white matter (*B* (95% CI): 0.5 (−20 to 21); *p* = 0.959), grey matter (*B* (95% CI): 2.6 (−16.1 to 21.3); *p* = 0.783), total brain (*B* (95% CI): 3.1 (−31.1 to 37.4); *p* = 0.857), or WMH volumes (*B* (95% CI): 0.24 (−0.46 to 0.94); *p* = 0.498) between NPSLE patients with ischemic phenotype and patients with non-NPSLE. These results did not show large differences when additionally adjusted for hypertension, diabetes, smoking, BMI, and SLE duration (Supplementary Table 4).

**Fig. 3:**
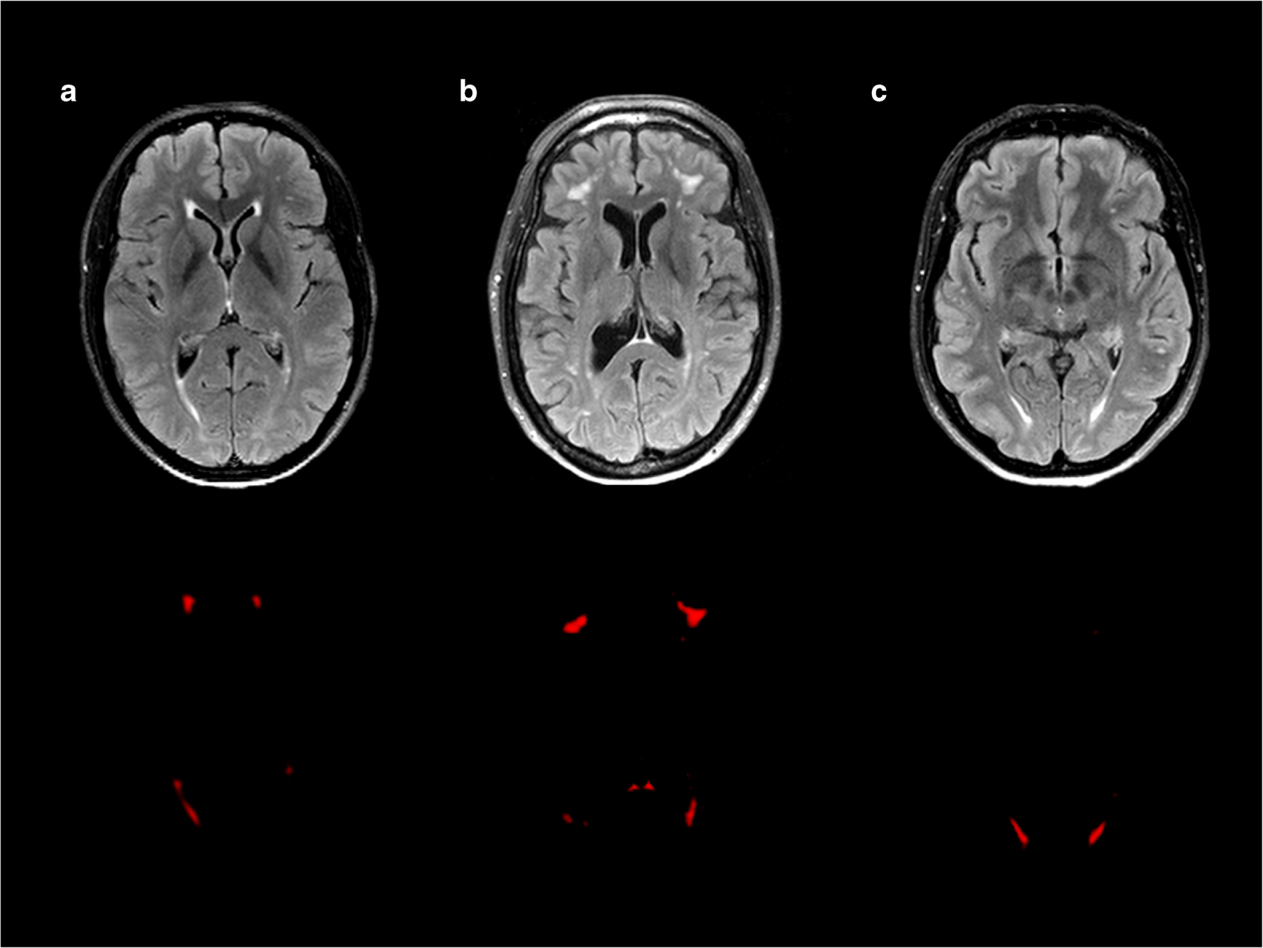
An example of white matter hyperintensities (WMH) in all groups of patients. The first row shows the WMH on the FLAIR brain MRI scans. The second row shows the WMH probability maps. **a** Non-neuropsychiatric systemic lupus erythematosus (non-NPSLE). Female, 37 years old. The MRI scan shows some periventricular and small deep WMH (total WMH volume: 3.04 ml). **b** Inflammatory neuropsychiatric systemic lupus erythematosus (inflammatory NPSLE). Female, 47 years old. The MRI scan shows deep and confluent WMH (total WMH volume: 18.52 ml) and cerebral atrophy. **c** Ischemic neuropsychiatric systemic lupus erythematosus (ischemic NPSLE). Female, 47 years old. The MRI scan shows some periventricular and deep WMH (total WMH volume: 3.31 ml)

NPSLE patients with inflammatory phenotype showed lower white matter (*B* (95% CI): −18.9 (−34.6 to −3.2); *p* = 0.020) and total brain volume (*B* (95% CI): −36.4 (−70.5 to −2.2); *p* = 0.038) compared to NPSLE patients with ischemic phenotype, but no differences in grey matter (*B* (95% CI): −17.5 (−42.5 to 7.5); *p* = 0.164) and WMH volume (*B* (95% CI): 0.66 (−0.34 to 1.65); *p* = 0.187) (see Table 4). These results did attenuate when analyses were additionally corrected for hypertension, diabetes, smoking, BMI, and SLE duration (Supplementary Table 4). Figure 3 shows an example of WMH in each patient group.

**Table 4:** Differences in brain volumes between the NPSLE patients (ischemic and inflammatory) and the non-NPSLE patients and between NPSLE ischemic and NPSLE inflammatory patients

	Non-NPSLE patients (*n* = 117)	NPSLE inflammatory patients (*n* = 26)	NPSLE ischemic patients (*n* = 12)	NPSLE inflammatory vs non-NPSLE (*B* (95% CI))	NPSLE ischemic vs non-NPSLE (*B* (95% CI))	NPSLE inflammatory vs NPSLE ischemic (*B* (95% CI))
White matter volume	482 ± 57	468 ± 56	485 ± 51	−17.1 (−31.4 to −2.8)*	0.5 (−20 to 21)	−18.9 (−34.6 to −3.2)*
Grey matter volume	560 ± 61	555 ± 69	564 ± 44	−13.7 (−27.6 to 0.3)	2.6 (−16.1 to 21.3)	−17.5 (−42.5 to 7.5)
Total brain volume	1042 ± 111	1022 ± 118	1049 ± 100	−30.7 (−55 to −6.4)*	3.1 (−31.1 to 37.4)	−36.4 (−70.5 to −2.2)*
WMH volume	0.60 (0.11–5.04)	1.06 (0.11–17.46)	1.11 (0.11–8.27)	0.87 (0.33 to 1.41)*	0.24 (−0.46 to 0.94)	0.66 (−0.34 to 1.65)

The second, third, and fourth columns represent volumes of white matter, grey matter, and total brain and white matter hyperintensity (WMH) volume of non-NPSLE, NPSLE inflammatory, and NPSLE ischemic patients in ml and these volumes are expressed as means ± standard deviations or as median (10–90% confidence intervals)

The fifth, sixth, and seventh columns represent *B* values (95% confidence interval) of the linear regression analysis on brain and WMH volumes respectively in NPSLE inflammatory patients vs non-NPSLE patients, in NPSLE ischemic patients vs non-NPSLE patients, and in NPSLE inflammatory patients vs NPSLE ischemic patients adjusted for gender, age, and total intracranial volume

For the linear regression analysis, the WMH were multiplied by 1,000,000 and natural log transformed, because of non-normal distribution

*p < 0.05

## Discussion

We showed that NPSLE patients had higher WMH volume compared to non-NPSLE patients, but no other brain volumes were significantly different between these groups. However, NPSLE patients with an inflammatory phenotype showed a lower total brain and white matter volume, and a higher WMH volume compared to non-NPSLE patients. Additionally, NPSLE patients with an inflammatory phenotype showed lower total brain volume and lower white matter volume compared to NPSLE patients with an ischemic phenotype.

For decades, structural MR imaging in NPSLE has mostly been used to exclude other diseases. Unfortunately, the link between apparent damage (lesions) and clinical symptoms remains weak (radiological-clinical paradox) [22]. However, in our study, we showed that the inflammatory phenotype of NPSLE shows a distinct pattern of brain changes, consisting of reduced total brain volume, reduced white matter volume, and increased WMH volume. Several previous studies assessed brain abnormalities (WMH and cerebral atrophy) in SLE patients compared to controls [5–9]. In particular, in case-control studies, SLE patients showed a decreased total brain volume [6] and a decrease in white matter and grey matter volumes [8] compared to age- and sex-matched controls. To the best of our knowledge, no previous studies have compared brain MRI volumes between different phenotypes of NPSLE. However, in other inflammatory diseases, such as multiple sclerosis, it has been shown that the inflammatory process is linked to cerebral atrophy and WMH [23]. We therefore hypothesized that in inflammatory NPSLE, continuous inflammation leads to brain atrophy and indeed observed that this phenotype is associated with more pronounced cerebral atrophy and more WMH.

Currently, differentiation between non-NPSLE, the ischemic phenotype of NPSLE, and the inflammatory phenotype of NPSLE based on clinical data alone can be challenging. This might lead to reestablishing the diagnosis further along the clinical course, based for example on response to treatment. Misdiagnosis can lead to suboptimal treatment or overtreatment [24]. The recognition of different patterns of brain changes on MRI, as demonstrated in our study, might help early in this diagnostic process to distinguish inflammatory NPSLE from non-NPSLE. Furthermore, our results might also help to further differentiate between the two NPSLE phenotypes. In particular, future diagnostic AI models to confirm NPSLE diagnosis might benefit from adding the markers that we have presented in our manuscript. Therefore, our results may lead to a faster and more accurate assignment of appropriate treatment regimens (glucocorticoids vs anticoagulants) in patients with a distinct NPSLE phenotype [25].

Besides the strengths of our study, namely, a well-defined cohort of SLE patients and the use of state-of-the-art MRI image processing routines, there are some limitations that need to be acknowledged. A limitation of our study could be the lower number of ischemic NPSLE patients compared to the other phenotypes. This was partly caused by the exclusion of NPSLE patients with large cerebral infarcts, because of errors in the brain segmentation. Moreover, even in our relatively large NPSLE patient population the prevalence of ischemic patients is low. Another limitation could be that as conventional MRI data are available at the time of multidisciplinary assessment, the difference in brain volumes between inflammatory NPSLE and non-NPSLE might be caused by circular reasoning: patients with a clinically apparent lower brain volume might be diagnosed with inflammatory NPSLE because of this. However, we checked all patient files and in only two patients with inflammatory NPSLE cerebral atrophy was reported on conventional MRI and might have contributed to the diagnosis. The potential bias introduced by this circular reasoning is therefore only small. Another limitation is the use of both 2D and 3D FLAIR MRI scans in our study, which may have introduced variations in measurements. To limit bias, our image processing pipeline consisted of methods that are relatively robust for differences in MRI sequences [26, 27]. The fact that our center is a tertiary referral center for NPSLE might limit the external validity of our results to the total NPSLE population as selective referral might have occurred. However, this bias is of less relevance for this study, as a (radiological) biomarker is especially needed for the most difficult NP presentations, which are in general referred to our center.

In conclusion, we showed that patients with NPSLE had a higher WMH volume compared to patients with non-NPSLE. Furthermore, especially the inflammatory phenotype of NPSLE was associated with the most pronounced volumetric brain changes. These findings may help in earlier and more accurate diagnosis of NPSLE.

## Supplementary information

ESM 1 (DOCX 34 kb)

## Abbreviations

FLAIR: Fluid-attenuated inversion recovery
NP: Neuropsychiatric
NPSLE: Neuropsychiatric systemic lupus erythematosus
SLE: Systemic lupus erythematosus
WMH: White matter hyperintensities

## Declarations

### Guarantor

The scientific guarantor of this publication is Dr. Jeroen de Bresser.

### Statistics and biometry

One of the authors has significant statistical expertise.

### Study subjects or cohorts overlap

Some study subjects or cohorts have been previously reported. The Department of Rheumatology at the Leiden University Medical Center is a national referral center for neuropsychiatric systemic lupus erythematosus. Over the last 20 years, the Departments of Rheumatology and Radiology have been engaged in a research partnership, aiming at improving the diagnosis of NPSLE patients and increasing the usefulness of various conventional and quantitative MRI modalities in understanding disease mechanisms and improving patient classification. This collaboration resulted in more than 20 independent publications, many of them using imaging data originating from the same cohort. Although imaging data of the same cohort has been used in various studies in this collaborative effort, this current study is our first advanced quantitative structural analysis of this patient cohort, and we attest here that the data we report in this study has not been reported in any way or form in any of our previous publications.

### Methodology

•Prospective

•Cross-sectional study

•Performed at one institution
