# Supplementary material for: Different phenotypes of neuropsychiatric systemic lupus erythematosus are related to a distinct pattern of structural changes on brain MRI
Source: Eur Radiol. 2021 Apr 30;31(11):8208–17. doi: 10.1007/s00330-021-07970-2 (PMC8523434; doi:10.1007/s00330-021-07970-2)
Supplement: Supplementary file 1 — (DOCX 34 kb) [file 330_2021_7970_MOESM1_ESM.docx]

SUPPLEMENTARY MATERIALS

Supplementary Table 1. NPSLE and non-NPSLE clinical variables.

|  | **non-NPSLE**  **(n = 117)** | **NPSLE**  **(n = 38)** |
| --- | --- | --- |
| ***Comorbidities*  *(n, %)*** |  |  |
| APS | 14 (12) | 6 (16) |
| ***Laboratory assessment (n,%)*** |  |  |
| Complement consumption | 44 (38) | 22 (58) |
| ANA | 81 (69) | 32 (84) |
| Anti-dsDNA^a^ | 25 (22) | 19 (54) |
| Anti-cardiolipin IgG/IgM ^b^ | 15 (13) | 6 (17) |
| Anti-B2-glycoprotein IgG/IgM ^c^ | 9 (8) | 5 (19) |
| LAC ^d^ | 29 (25) | 12 (32) |
| ***Medication (n, %)*** |  |  |
| Corticosteroids | 57 (49) | 27 (71) |
| Antiplatelet | 19 (16) | 11 (29) |
| Anticoagulant | 9 (8) | 4 (11) |
| ***Phenotype (n, %)*** |  |  |
| Inflammatory | - | 26 (68) |
| Ischemic | - | 12 (32) |
| ***NPSLE syndromes***  ***(n, % of total syndromes)*** | * |  |
| Aseptic meningitis | - | 1 (3) |
| Cerebrovascular disease | - | 11 (29) |
| Demyelinating syndrome | - | 0 (0) |
| Headache | - | 3 (8) |
| Movement disorder | - | 2 (5) |
| Myelopathy | - | 3 (8) |
| Seizure disorder | - | 3 (8) |
| Acute confusional state | - | 4 (11) |
| Anxiety disorder | - | 0 (0) |
| Cognitive dysfunction | - | 11 (29) |
| Mood disorder | - | 6 (16) |
| Psychosis | - | 0 (0) |
| AIDP | - | 0 (0) |
| Autonomic disorder | - | 0 (0) |
| Myasthenia gravis | - | 0 (0) |
| Cranial neuropathy | - | 3 (8) |
| Mononeuropathy | - | 0 (0) |
| Polyneuropathy | - | 2 (5) |
| Plexopathy | - | 0 (0) |
| Other ^e^ | - | 4 |

Laboratory assessment available for ^a^ 113/117 and 35/38, ^b^ 36/38 NPSLE patients, ^c^  79/117 and 26/38, ^d^  116/117 and 35/38 patients.

*AIDP =*  *acute inflammatory demyelinating polyradiculoneuropathy, ANA = antinuclear antibodies, anti-dsDNA = anti-doublestranded DNA, APS = antiphospholipid syndrome, LAC = lupus anticoagulans*

^e^ Other NPSLE: organic brain syndrome (n = 2), walking disorder (n = 1), lethargia (n = 1)

Supplementary table 2. NP events in non-NPSLE patients.

|  | Non-NPSLE  (n = 117) |
| --- | --- |
| ***Diagnosis/symptom (n, % of total)*** |  |
| Mood disorder | 28 (16) |
| Anxiety disorder | 4 (2) |
| Psychiatric disorders, other^a^ | 8 (4) |
|  |  |
|  |  |
| Headache | 45 (25) |
| Cognitive complaints |  |
| *Objective* | 17 (9) |
| *Subjective* | 20 (11) |
|  |  |
| Sensibility disorder |  |
| *Polyneuropathy* | 2 (1) |
| *Objective (other)* | 3 (2) |
| *Subjective* | 3 (2) |
| Cerebrovascular disease | 3 (2) |
| Epilepsy | 8 (4) |
| Dizziness/ collapse | 11 (6) |
| Neurological disorders, other^b^ | 11 (6) |
|  |  |
| Side-effects of medication | 8 (4) |
| Malignancy | 1 (1) |
| Delirium | 1 (1) |
| Other^c^ | 6 (3) |
|  |  |

This table represents diagnoses and symptoms (a total of 179) in patients with non-NPSLE (n = 117).

^a^ Other psychiatric diagnoses include, amongst others: dissociative disorder, personality disorder, PTSD, coping disorder

^b^ Other neurological diagnoses include, amongst others: hernia nucleus pulposi, carpel tunnel syndrome, dementia

^c^ Other disorders include, amongst others: fatigue, Whiplash, sleeping disorder

Supplementary table 3- Differences in brain volumes between the NPSLE patients and the non-NPSLE patients (corrected for gender, age, total intracranial volume, diabetes, hypertension, smoking, BMI and SLE duration).

|  | **NPSLE patients (n=38)** | **Non-NPSLE patients (n=117)** | **NPSLE vs non-NPSLE**  **(B (95%-CI))** |
| --- | --- | --- | --- |
| **White matter volume** | 473±55 | 482±57 | -11 (-23.1 to 1.2) |
| **Grey matter volume** | 557±62 | 560±61 | -8.6 (-20.6 to 3.4) |
| **Total brain volume** | 1030±110 | 1042±111 | -19.6 (-40.4 to 1.2) |
| **WMH volume** | 1.08 (0.12–14.94) | 0.60 (0.11–5.04) | 0.55 (0.10 to 1.0)* |

The second and third column represent white matter, grey matter, total brain and white matter hyperintensity (WMH) volume in ml expressed as means ± standard deviations or median (10-90% confidence intervals).

The fourth column represents B (95%-confidence intervals) of the linear regression analyses on brain and WMH volumes in NPSLE patients versus non-NPSLE patients, adjusted for gender, age, total intracranial volume, diabetes, hypertension, smoking, BMI and SLE duration. For the linear regression analysis the WMH were multiplied times 1.000.000 and natural log transformed, because of non-normal distribution.

* p<0.05

Supplementary table 4- Differences in brain volumes between the NPSLE patients (ischemic and inflammatory) and the non-NPSLE patients and between NPSLE ischemic and NPSLE inflammatory patients (corrected for gender, age, total intracranial volume, diabetes, hypertension, smoking, BMI and SLE duration).

|  | **Non-NPSLE patients (n=117)** | **NPSLE inflammatory patients**  **(n=26)** | **NPSLE**  **Ischemic patients**  **(n=12)** | **NPSLE inflammatory vs non-NPSLE**  **(B (95%-CI))** | **NPSLE ischemic vs non-NPSLE**  **(B (95%-CI))** | **NPSLE inflammatory vs NPSLE ischemic**  **(B (95%-CI))** |
| --- | --- | --- | --- | --- | --- | --- |
| **White matter volume** | 482±57 | 468±56 | 485±51 | -16.5 (-31.2 to -1.7)* | 0.4 (-20.1 to 20.8) | -10.8 (-29.4 to 7.8) |
| **Grey matter volume** | 560±61 | 555±69 | 564±44 | -14.8 (-29.1 to -0.5)* | 2.9 (-15.5 to 21.3) | -12.5 (-42 to 17.1) |
| **Total brain volume** | 1042±111 | 1022±118 | 1049±100 | -31.3 (-56.2 to -6.5)* | 3.2 (-30.3 to 36.8) | -23.2 (-64.5 to 18.1) |
| **WMH volume** | 0.60 (0.11–5.04) | 1.06 (0.11–17.46) | 1.11 (0.11–8.27) | 0.72 (0.17 to 1.26)* | 0.22 (-0.46 to 0.91) | 0.19 (-0.88 to 1.27) |

The second, third and fourth column represent volumes of white matter, grey matter, total brain and white matter hyperintensity (WMH) volume of non-NPSLE, NPSLE inflammatory and NPSLE ischemic patients in ml and expressed as means ± standard deviations or as median (10-90% confidence intervals).

The fifth, sixth and seventh column represent B values (95%-confidence interval) of the linear regression analysis on brain and WMH volumes in NPSLE inflammatory patients vs non-NPSLE patients, in NPSLE ischemic patients vs non-NPSLE patients and in NPSLE inflammatory patients vs NPSLE ischemic patients. These analyses were adjusted for gender, age, total intracranial volume, diabetes, hypertension, smoking, BMI and SLE duration.

For the linear regression analyses the WMH were multiplied times 1.000.000 and natural log transformed, because of non-normal distribution.

* p<0.05
